# Manipulating Pico- to Nanoliter Droplets on Surfaces without Sticking

**DOI:** 10.1021/acsnano.5c14919

**Published:** 2025-11-06

**Authors:** Mizuki Tenjimbayashi, Shunto Arai, Hiroshi Mizoguchi, Satoshi Ishii

**Affiliations:** † Research Center for Materials Nanoarchitectonics (MANA), National Institute for Materials Science (NIMS), 1-1 Namiki, Tsukuba, Ibaraki 305-0044, Japan; ‡ Research Center for Macromolecules and Biomaterials, National Institute for Materials Science (NIMS), 1-1 Namiki, Tsukuba, Ibaraki 305-0044, Japan

**Keywords:** nanomicrometer hierarchical particles, nonsticking picoliter droplet, liquid marble, superomniphobicity, droplet manipulation

## Abstract

Droplet manipulation on surfaces is ubiquitous in many industrial fields. Liquid-repellent surfaces are required to facilitate manipulation because sticking restricts droplet motion. Various liquid-repellent surfaces have been used to manipulate microliter droplets. However, classical surfaces suffer from the repellence of pico- to nanoliter droplets. This study demonstrates the nonsticking property of pico- to nanoliter droplets on surfaces when it is coated with low-surface-energy particles with nano–micrometer hierarchy. The dynamic particle coating of ultrasonic-sprayed droplets enables the formation of highly spherical, isolated, particle-coated picoliter droplets. The particle coating changes the solid–liquid interfacial friction to solid–solid interfacial friction and reduces the force required to move the droplet to the subnanonewton range. Consequently, picoliter droplets slide off a tilted substrate without sticking. The coating does not affect the fluid shape reconfigurability of the droplets. This approach facilitates diverse and complex multiway manipulation of picoliter droplets, allowing separation, arrangement, transportation, and shape reconfiguration without sticking. This advances the understanding of droplet behavior at interfaces, and the proposed method may contribute to downsizing fluidic systems.

## Graphical abstract

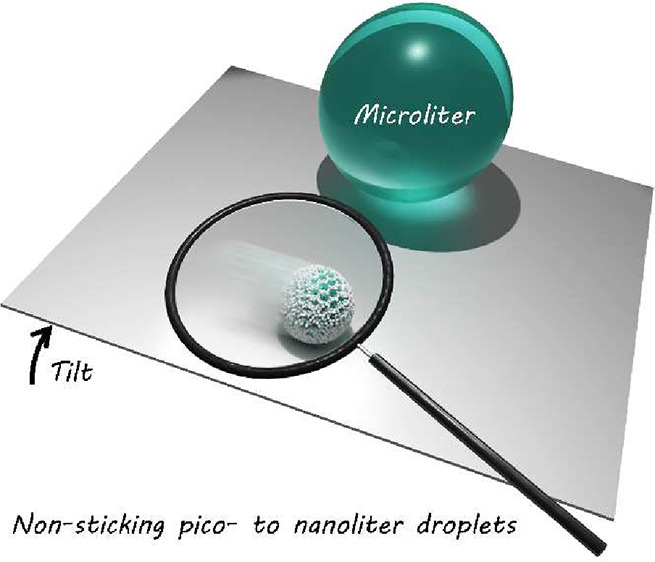

## Introduction

The manipulation of droplets on surfaces is essential across numerous industrial fields.^1–9^ For example, precise handling of progressively smaller volumes of liquids is increasingly important for droplet microfluidics applications such as chemical reactors,^10–12^ bioanalysis,^13,14^ and cargo delivery.^15–17^ Liquid-repellent surfaces have been suggested as a powerful platform for microliter droplet manipulation because sticking is a serious problem for small-volume droplets. Traditionally, various liquid-repellent surfaces have been formed on droplet-contacting substrates. Representative surfaces such as hydrophobic, superhydrophobic, superomniphobic, lubricant-impregnated, and liquid-like surfaces have successfully repelled microliter droplets.^18–22^ However, they are less effective with smaller droplets, which are more sensitive to the chemotopological heterogeneity of the surface, and the reduction of solid–liquid interfacial friction is a limiting factor.^23–28^ Therefore, a trade-off exists between droplet sticking and droplet volume. The droplet friction on these surfaces limits the minimum repellable volume to the order of microliters (Table S1).^23–26,19,20,22,29–34^ Despite previous efforts,^10,35–41^ imperfect and nongeneral nanoliter droplet repellence limits the diversity and complexity of droplet manipulation. Exceptionally, Leidenfrost droplets—i.e., droplets floating on a heated substrate—exhibit negligible friction.^42–44^ However, severe limitations in substrate/droplet combinations and high energy consumption restrict their practical use.

Alternatively, liquid-repellent structures have been formed on droplet surfaces by coating them with low-surface-energy nanometer and/or micrometer particles. The particle-coated millimetric droplets exhibit nonsticking properties on surfaces and are recognized as liquid marbles.^45^ Unlike bulk-film-coated droplets, particle-coated droplets retain their fluid shape reconfigurability. The dynamics of particle-coated droplets (liquid marbles) have been investigated.^46–48^ Recent studies have suggested that liquid marble friction is constantly low at the microliter scale, regardless of the droplet volume.^32^ However, liquid marble friction at smaller scales has not been investigated thoroughly and the minimum volume where particle-coated droplets exhibit low friction is unknown. This is because the formation of spherical and isolated liquid marbles at pico- to nanoliter volume is challenging while recent work has achieved a minimum volume of liquid marble of approximately 0.2 μL.^49^

In this study, we developed our original and broadly applicable technique^50^ for the preparation of isolated high-sphericity particle-coated picoliter droplets (see Coating Droplets with Superomniphobic Particles). We find the particle-coated droplets exhibited nonsticking properties, even at picoliter volumes. The nonsticking property offered diverse and complex multiway manipulation of picoliter droplets including arrangement, transport, switchable sticking, and shape reconfiguration.

## Results and Discussion

### Nonsticking Property of Particle-Coated Droplets

Figure 1a–c show the droplet size-dependent sticking properties when either the droplet or substrate was coated with liquid-repellent particles. The liquid-repellent particles used in this study were fluorocarbon-modified fumed titania particles with a primary diameter of ∼20 nm and single-micrometer aggregation. Owing to the fluorination, the particles had a low critical surface tension of 26 ± 1 mN/m (Figure S1).

**Figure 1:**
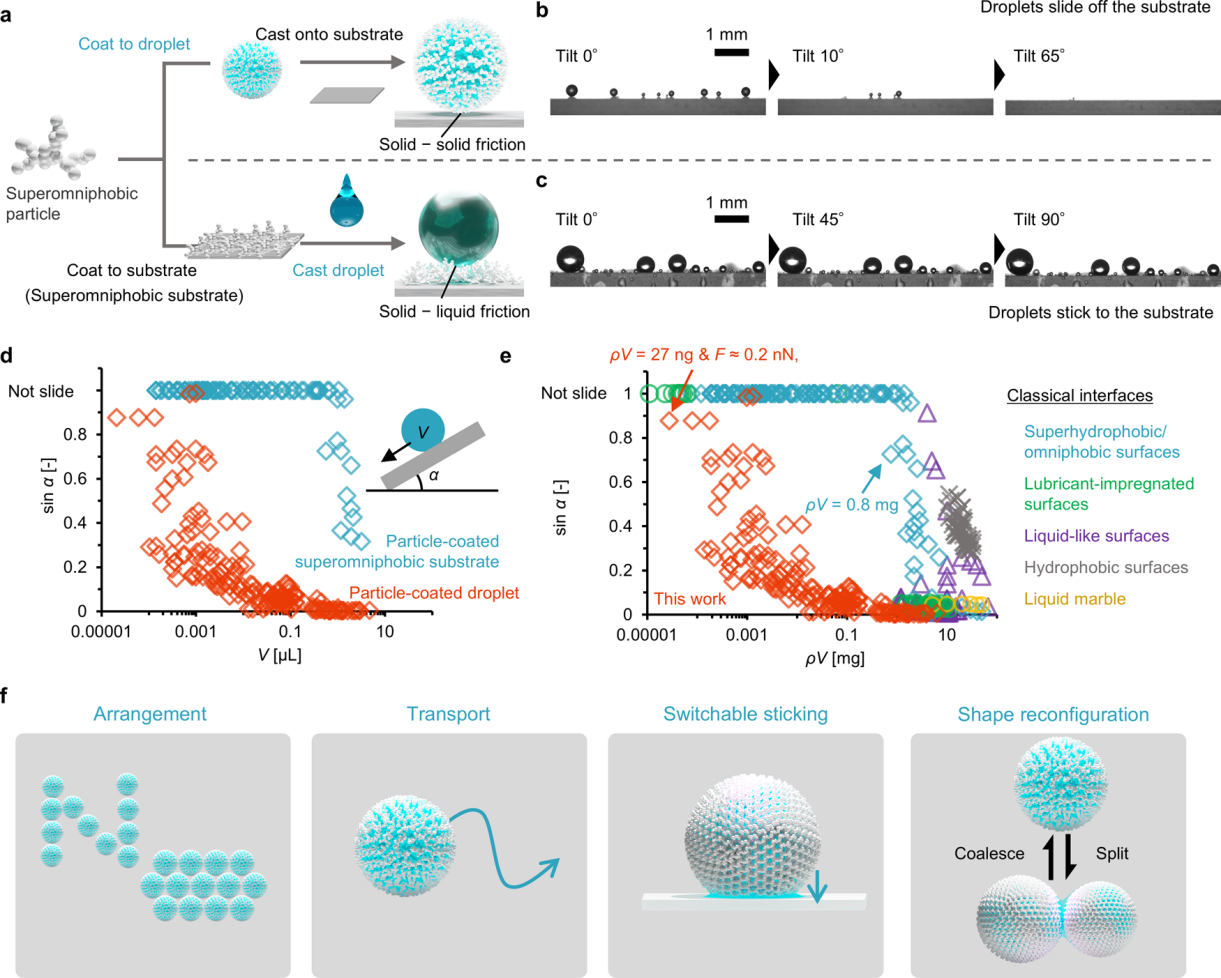
Picoliter droplet repellence using superomniphobic particles. (a) Sliding behavior of picoliter droplets when the droplets (top) and substrate (bottom) were coated with superomniphobic particles. Droplet sliding behavior when the substrate was tilted at 0.1°/s and the (b) droplets and (c) substrate were coated with particles. Scale bars: 1 mm. Effect of droplet volume *V* on the sliding angle α for (d) particle-coated droplets and substrate and (e) various liquid-repellent surfaces. The horizontal axis in (e) shows the droplet volume multiplied by the liquid density to allow the sliding angle to be compared without considering the effects of liquid density. The plot shows improvement of the proposed method compared to those reported previously (Table S1 for details). (f) Schematic of the micrometer-sized droplet manipulations achieved in this study.

The particle-coated substrate showed nano–micrometer hierarchical structures with low surface energy, and it exhibited excellent liquid repellency, known as superomniphobicity. For a volume *V* of 10 μL (diameter ≈ 2.7 mm), the substrate had an apparent static contact angle θ of 171 ± 1° for water (surface tension ≈ 72 mN/m) and 164 ± 1° for oleic acid (surface tension ≈ 33 mN/m) (Figure S2). In this study, 1-(2-hydroxyethyl)-3-methylimidazolium tetrafluoroborate, which has surface tension ≈ 58 mN/m and liquid density ρ ≈ 1340 kg/m^3^, was used as the liquid for the probe droplet to minimize the effects of droplet evaporation. Sticking was assessed based on the critical substrate tilting angle α which caused the droplet to slide off the substrate. In this case, the droplet friction *F* is equal to the component of the gravitational force parallel to the tilted substrate, *F* ≈ ρ*Vg* sin α*,* where *g* is the acceleration due to gravity. We assumed that the mass of the particles on the droplet was negligible compared to the mass of the droplet because the tamped density of the particles (∼10^2^ kg/m^3^) was much less than the density of the liquid (∼10^3^ kg/m^3^) and the thickness of the particle layer (∼1 μm) was much less than the diameter of the droplet (Figure S3).

When uncoated droplets were cast on the superomniphobic particle-coated substrate, α was small for millimetric droplets and increased exponentially as the droplet volume decreased (Figure 1c,d). We estimated that sin α ∼ *V* ^–2/3^ (see Note S1 and Figure S4 for the discussion). When particle-coated droplets were cast on the substrate, α increased exponentially as the droplet volume decreased. However, compared to the case with the particle-coated substrate, much smaller particle-coated droplets were able to slide off the substrate (Figure 1a,b,d and Movie S1). These differences occurred owing to the differences between droplet–particle interactions in the former case and particle–substrate interactions in the latter case (see Note S2 and Figure S5 for the discussion). The observed minimum *F* for the particle-coated droplet was approximately 0.2 nN for *V* ≈ 20 pL or ρ*V* ≈ 27 ng, which is of the same order as colloidal forces originating from van der Waals interactions.^51^

We compared the volume-dependent sliding angles of particle-coated droplets and classical liquid-repellent interfaces (Figure 1e and Table S1) and found that the proposed approach reduced the droplet-repellent volume by three to four orders of magnitude. This reduction in the liquid-repellent volume presents opportunities for nonsticking picoliter droplet manipulation, which we explored in this study (Figure 1f). The following sections demonstrate how diverse and complex manipulation of picoliter droplets can be achieved using the particle-coating strategy.

### Coating Droplets with Superomniphobic Particles

The setup used to obtain superomniphobic particle-coated droplets at picoliter volume is shown in Figure 2a. Picoliter droplets of the ionic liquid, whose diameter is tens micrometer, were produced using an ultrasonic spray and impacted on a superomniphobic particle powder bed under vibration. The picoliter droplets can also be produced using a commercially available hand sprayer (Figure S6). Furthermore, heating liquids or commercial mist diffusers may also be viable methods of producing picoliter droplets. The particles were adsorbed on the droplet surface rather than diffusing inside because the critical surface tension of the particles was significantly smaller than that of the droplet and the particle exhibited low wettability to the droplet.^52^ When ethanol (surface tension ≈ 22 mN/m) or liquid polydimethylsiloxane (PDMS) (surface tension ≈ 20 mN/m) is used as the core liquid, particle-coated droplets are not formed, instead we observed powder agglomeration (Figure S7).

**Figure 2:**
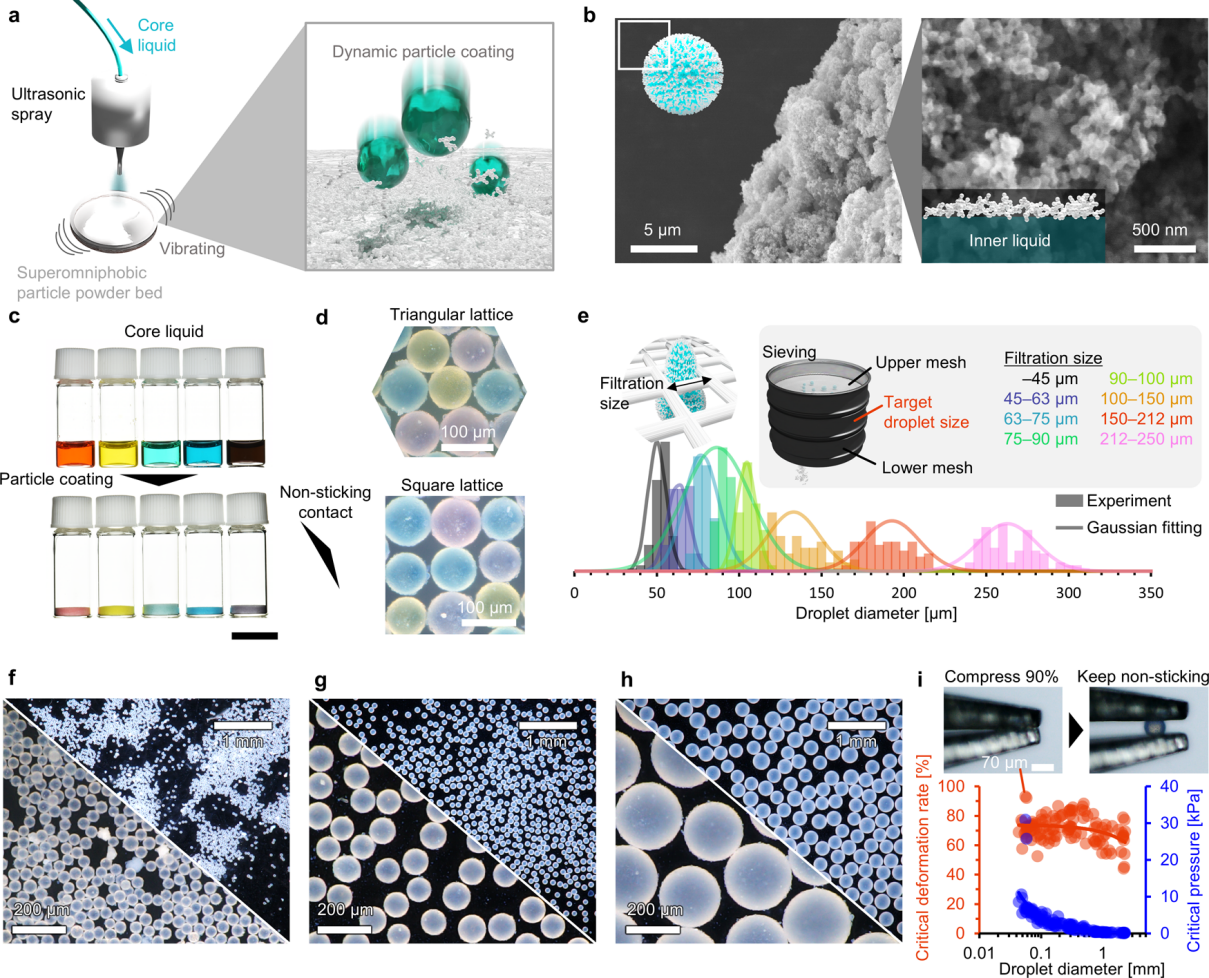
Coating droplets with superomniphobic particles. (a) Schematic of the method used to coat the picoliter droplets with superomniphobic particles. (b) SEM images of the superomniphobic particle-coated droplets. Scale bars: 5 μm (left) and 100 nm (right). (c) Photographs of the core liquid (top) and coated droplets (bottom) in glass bottles. Scale bars: 5 mm. (d) Nonsticking contact of the droplets. Triangular and square lattice packings were observed. Scale bars: 100 μm. (e) Droplet size was controlled by sieving. (f–h) Microscopy images of droplets with different sizes. Scale bars: 200 μm and 1 mm for higher and lower magnification images, respectively. (i) Deformation stability of the droplets as a function of the droplet diameter during compression. Scale bar: 70 μm.

Fluorine-free hydrocarbon-modified particles also adsorbed to the droplet surface to form particle-coated droplets at picoliter volume (Figure S7). Our previous study revealed that the nanomicro hierarchy is crucial to form small liquid marble.^50^ However, although commercial hydrocarbon-modified fumed titania particles can be used to produce particle-coated droplets at microliter volume, they cannot be used with picoliter droplets owing to powder agglomeration (Figure S7). Therefore, the quality of the modification/aggregation is an important consideration to produce particle-coated droplets at picoliter volume.

Particle adsorption produced a dynamic particle coating on the droplet and its structure was observed using field-emission scanning electron microscopy (SEM) (Figure 2b). Owing to the nonvolatility of the core liquid, direct SEM observations of the particle-coated droplets were possible. The particle layer was approximately 1 μm thick and exhibited a hierarchical structure of 20 nm primary particles and ∼2 μm aggregates. We prepared particle-coated droplets of different colors by dying the core liquid and copowdering them on a glass substrate (Figure 2c).

When the particle-coated droplets were in contact with each other (Figure 2d), the contact area formed a solid–solid interface owing to the particle coating, which prevented them from sticking and coalescing and stable for at least 30 d in a sealed glass bottle (Figure S7). We observed that the droplets were closely packed into triangular or square lattices, similar to granules or colloids, depending on their density. Because the droplets did not stick to each other, sieving was used to sort the droplets by diameter. The diameter distributions (Figure 2e) were mostly limited to the upper and lower mesh pore sizes, and highly uniform droplets of different sizes were obtained by dispersing them in air (Figures 2f–h and S8). However, the upper limit of the filtered droplet size slightly exceeded the filtration size, which was probably due to droplet deformation during filtration. It means droplets retained their nonsticking properties even in the deformed state, which was confirmed by the compression stability test (Figure 2i). The critical deformation rate for the droplet nonsticking property increased as the droplet diameter decreased. Furthermore, the critical compression pressure, estimated from the Laplace pressure,^53^ increased as the droplet size decreased. We found that a droplet with a diameter of approximately 100 μm retained its nonsticking properties even after 90% compression (Movie S2). This robust nonsticking property helps to facilitate the droplet handling in sorting.

### Arrangement and Transport

Owing to their nonsticking properties and compression stability, the droplets behaved like a powder; that is, in addition to piling on solid substrates, they could also pile on a water pool owing to their water repellence (Figure 3a and Movie S3). In the movie, the droplets appear to move without sticking on both these surfaces because they are not wet.

**Figure 3:**
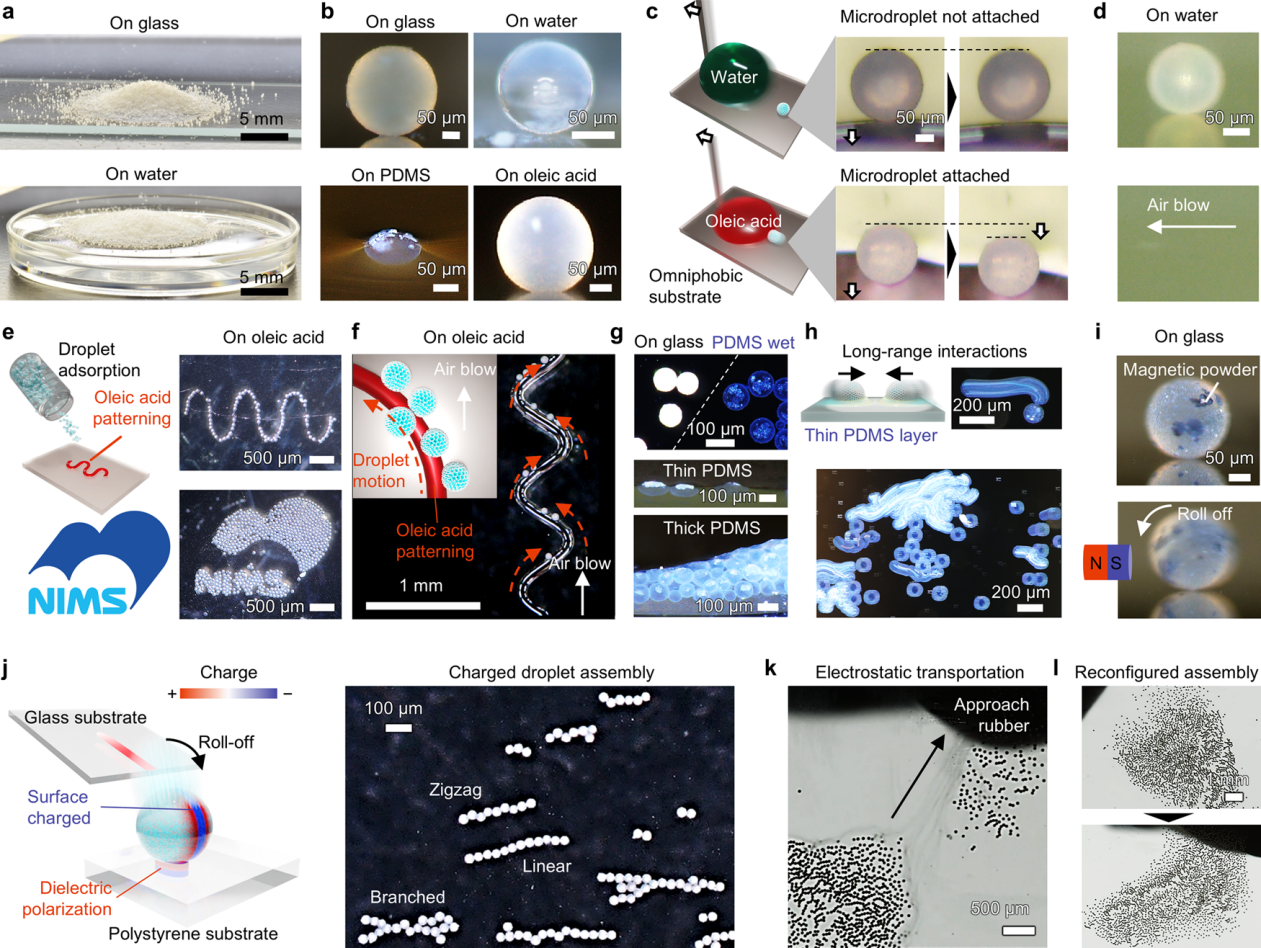
Arrangement and transport. (a) Particle-coated droplets lie thick on glass and water surfaces like powder. The advancing angles of the repose were approximately 26 and 15° on glass and water, respectively. Scale bars: 5 mm. (b) Side views of droplets on various surfaces; θ denotes the apparent contact angle. Scale bars: 50 μm. (c) Sticking behavior of droplets on water or oleic acid. Scale bar: 50 μm. (d) Droplet transportation on water controlled by airflow. Scale bar: 50 μm. (e) Patterned droplet deposition. Oleic acid patterns were produced, and droplets were deposited on the oleic acid surface, enabling on-demand arrangement of the droplets. Scale bars: 500 μm. (f) Stuck image of preprogrammed droplet transportation. The droplets moved parallel to the oleic acid surface; thus, the motion orbital drew a patterned oleic acid contact line shape. Scale bar: 1 mm. (g) Behavior of the droplets in PDMS. Opaqueness of the droplets before and after lubrication (top). Side views of the droplets partially sunk in a thin PDMS layer (middle) and dispersed in thick a PDMS layer like an emulsion (bottom). Scale bars: 100 μm. (h) Long-range interactions of droplets cloaked in a thin PDMS layer. Motion orbitals of droplets expressed by stuck-in-time images. Scale bars: 200 μm. (i) On-demand transportation of droplets doped with Sr ferrite using a magnet. Scale bar: 50 μm. (j–l) Assembly of charged picoliter droplets. Contact electrification between the glass substrate and fluorinated nanoparticles electrostatically charged the contact region of the droplets. (j) One-dimensional assembly of the partially charged droplets on a polystyrene dish. Scale bars: 100 μm. (k) Electrostatic collective transportation of the charged droplets. Scale bar: 500 μm. (l) Electrostatic reconfiguration of the droplet assembly. Scale bar: 1 mm.

The shapes of the droplets on glass and various liquid surfaces were shown in Figure 3b. The apparent contact angles of the droplets were used to quantify the droplet adhesion to the liquid surfaces. This indicated that the adhesion of the droplets depended on the liquid. They sat on the surface of the water and appeared almost perfectly spherical, with a contact angle of 176 ± 2°, such that no clear water–droplet contact was visible in the side view photo. By contrast, the droplets on the oleic acid surface were not perfectly spherical, with a contact angle of 167 ± 4°, and oleic acid–droplet contact was apparent. Therefore, the droplets showed different adhesion behavior with water and oleic acid. As shown in Figure 3c, a picoliter droplet attached to a large water or oleic acid droplet was slid on an omniphobic substrate. The picoliter droplet did not follow the water droplet because the adhesion was negligible. By contrast, it followed the oleic acid droplet, which indicates that the picoliter droplets adhered to the oleic acid surface.

The difference in adhesion properties of picoliter droplets on solid and liquid surfaces presents ideas for manipulating picoliter droplets. Air could be used to blow them off a water surface owing to their low adhesion (Figure 3d and Movie S4). By contrast, oleic acid could be used to arrange them in a patterned shape owing to their adhesion (Figure 3e). In this state, the freedom of motion of the picoliter droplets was limited to the direction parallel to the oleic acid surface, and the droplets moved along the shape of the oleic acid surface. Thus, the motion of the droplets was preprogrammed by the patterned deposition of oleic acid (Figure 3f and Movie S5).

We observed the droplets sunk into the liquid PDMS with an apparent contact angle of 59 ± 2° (Figure 3b). Here, we investigated the droplets impregnated into liquid PDMS (Figure 3g,h and Movie S6). After impregnation, two scenarios were observed. In the first scenario, the air between the particles in the droplet coating was replaced by PDMS, which was confirmed by the optical changes in the droplets (see the top image in Figure 3g). Consequently, the droplets were cloaked in a thin layer of PDMS with a thickness comparable to the diameter of the droplets (see the middle image in Figure 3g). In the second scenario, the droplets were wrapped in a thick layer of PDMS, where the surrounding droplets were replaced by PDMS, as in emulsions (see the bottom image in Figure 3g). Here, the droplet density and size could be controlled independently by controlling the PDMS-to-droplet ratio and sieving mesh size, respectively. The droplets cloaked in a thin layer of PDMS exhibited collective motion (Figure 3h and Movie S6). We observed interactions between the droplets, and the swarm exhibited complex collective self-propelling motion. This was related to the capillary force of the PDMS because the cloaked droplets distorted the PDMS surface (Figure 3b) and affected the direction of the capillary force, which is a known phenomenon in uncoated droplets.^38,54^ Another strategy for driving the droplets involved doping them with magnetic powder (Figure 3i and Movie S7). Owing to their magnetic field responsivity, the doped droplets exhibited magnetically regulated on-demand motion.

Because the droplets behaved like colloidal particles, they could be assembled by surface charging. We rolled the droplets off a glass substrate and dropped them onto a polystyrene substrate (Figure 3j). Owing to their relative positions in the triboelectric series, the glass surface was positively charged and the fluorinated particles on the droplets were negatively charged through triboelectrification. The charged region was limited to the contact area between the droplets and the glass substrate; therefore, the droplets dropped onto the polystyrene substrate had a biased surface charge distribution. Consequently, the region of the droplet surface near the border of the charged region and the region in contact with the polystyrene substrate were positively polarized, which resulted in a soft arrest at the substrate (Movie S8). Moreover, the biased droplet charge resulted in a one-dimensional assembly of the droplets (Figure 3j). Furthermore, collective directional manipulation of the droplets and droplet assembly reconfiguration were achieved by approaching the polystyrene substrate with a rubber surface (Figure 3k,l and Movie S8). The prototype flexible droplet motion and deposition demonstrated in Figure 3 suggest that various strategies can be used to manipulate picoliter droplets without sticking.

### Switchable Sticking and Shape Reconfiguration

Unlike continuous film-coated droplets, particle-coated droplets maintain fluid shape reconfigurability through wetting, coalescing, or splitting by external stimuli (Figure 4). We focused on the photocatalytic activity of the particles. X-ray Rietveld refinement showed that the titania particles consisted of anatase (∼91%) and rutile (∼9%) phases (Figure S9). The particles became omniphilic after energy input because the anatase crystal acted as a photocatalyst.^55,56^ As expected, the omniphilized particles detached from the liquid surface (Figure 4a), which was confirmed by SEM. The liquid surface was exposed when the electron beam focused on a particle-coated droplet inside the SEM chamber. At the border of the exposed liquid, the particles were partially wetted by the core liquid, which was consistent with our expectations.

**Figure 4:**
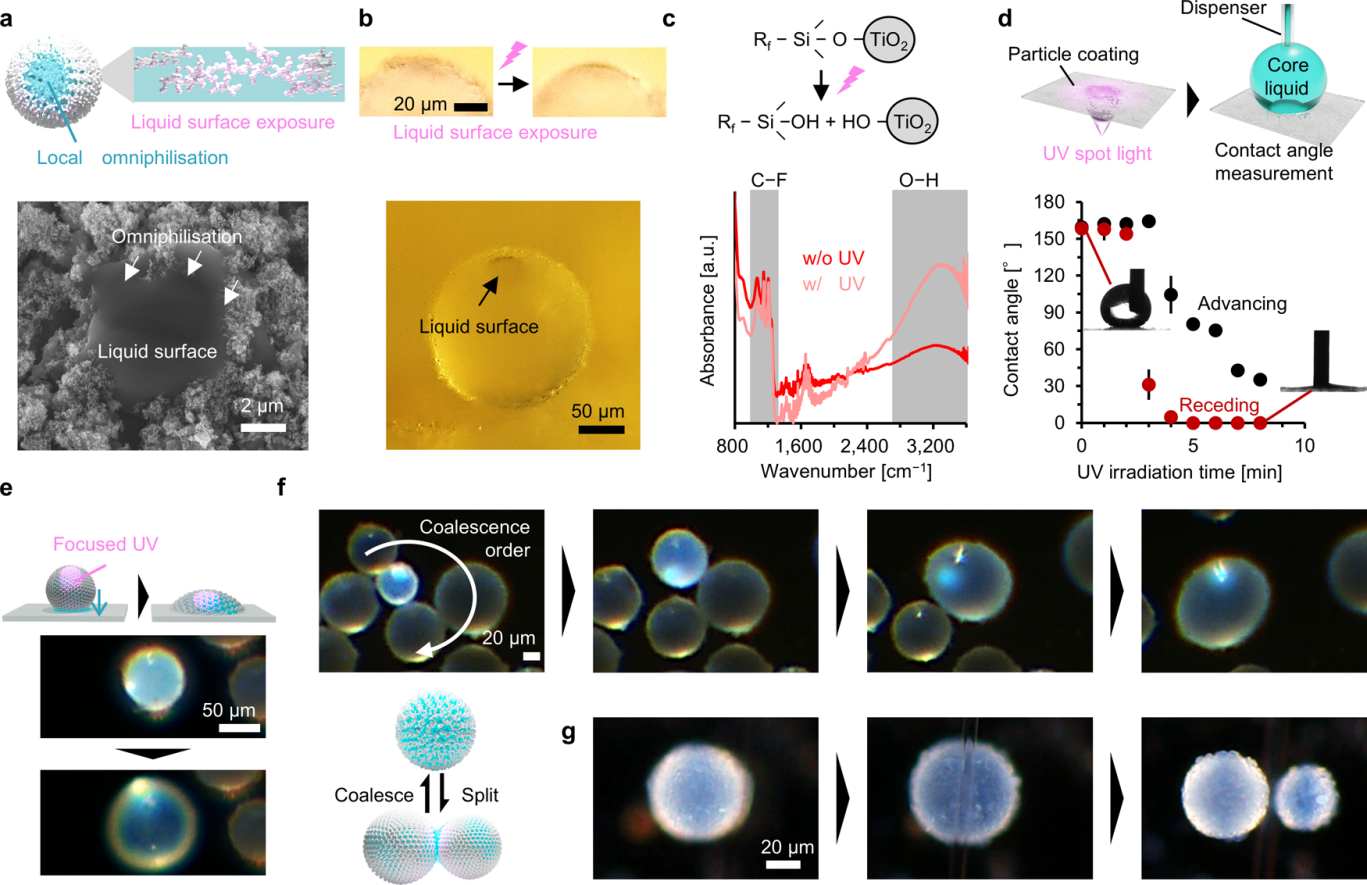
Switchable sticking and shape reconfiguration. (a) Stimuli-responsive local omniphilization of particle-coated droplets. Ionic liquid surfaces were exposed to focused UV irradiation during omniphilization, which was confirmed by field-emission SEM. Scale bar: 2 μm. (b) Observation of the liquid surface exposed by UV irradiation. Top two images: the variation in the droplet’s outline shape when the liquid surface was exposed by irradiated UV. Scale bar: 20 μm. Bottom image: selective exposure of the liquid surface by focused UV laser irradiation. Scale bar: 50 μm. (c) Omniphilization was induced by photocatalytic detachment of the omniphobic molecules from the particle, which was confirmed by FT-IR spectroscopy before and after UV irradiation. (d) Variations in UV-induced particle wettability confirmed by the dynamic contact angles on a particle-coated glass substrate after different UV irradiation times. (e) Switch to the sticking state of the droplets triggered by a focused UV laser. Scale bars: 50 μm. (f) On-demand cascade coalescence of the droplets triggered by a UV laser. Scale bars: 20 μm. (g) Droplets mechanically split by cutting with a glass needle. Scale bars: 20 μm.

Although exposure of the liquid surface can facilitate shape reconfiguration, electron beams cannot be used in air. Therefore, we used ultraviolet (UV) light, which is absorbed by the particles, to expose the liquid surface instead (Figure S10). The liquid surface exposure was confirmed by direct observation using digital microscopy (Figure 4b). Upon UV irradiation, the particle layer had detached from the liquid surface. UV-induced omniphilization occurred because the fluorocarbon groups detached from the particles. This was confirmed by Fourier Transform Infrared (FT-IR) Spectroscopy, in which the intensity of O–H stretching peak increased and C–F peaks decreased after UV irradiation (see Figures 4c and S11 for the FT-IR peaks of the unmodified particles). Omniphilization occurred owing to the change in surface chemistry because the structure of the particles was not affected by UV irradiation (Figure S12). The omniphilization was quantified based on the contact angle of the core liquid on the particle-coated substrate (Figure 4d), which indicated that both the advancing and receding contact angles decreased as the UV irradiation time increased.

Exposure of the liquid in the nonsticking droplets resulted in stimuli-responsive shape reconfigurability. When the bottom of a droplet was irradiated with UV light, it adhered to the substrate (Figure 4e and Movie S9). Moreover, UV irradiation of neighboring droplets enabled selective coalescence (Figure 4f and Movie S10). Furthermore, the droplets could be split mechanically using a thin glass rod (Figure 4g and Movie S11). Owing to the particle layer, split droplets do not coalesce without external stimuli. The droplet splitting enables the separation of inner liquid components. The cyclic coalescence and splitting of droplets may diversify droplet microfluidics.

## Conclusions

Our findings offer insights into the manipulation of picoliter droplets without sticking by particle-coating. The variations in the possible functional droplet/particle combinations and manipulation strategies present opportunities for applications such as sticking-free pico-/nanofluidics and soft microrobotics, which has recently been considered for particle-coated droplets at microliter scale.^57–60^ Moreover, the colloidal particle-like collective behavior of the droplets—including electrostatic and lubricated assembly—may advance the swarm droplet systems. Such collective motion is challenging for millimetric droplets because they are dominated by gravitational and hydrodynamic forces rather than thermal fluctuations or interdroplet interactions. Therefore, we believe this work offers new approaches that bridge droplet transport and colloidal self-assembly.

## Methods

### Materials

All the materials were used as received. Fumed titania particles (AEROXIDE TiO_2_ P 90) and commercial alkyl-modified fumed titania particles (AEROXIDE TiO_2_ NKT90) were obtained from Evonik Industries (Germany). 1H,1H,2H,2H-perfluorotrichlorosilane was obtained from Angene International Ltd. (China). Methyl trichlorosilane was obtained from Tokyo Chemical Industry Co., Ltd. (Japan). The ionic liquid, 1-(2-hydroxyethyl)-3-methylimidazolium tetrafluoroborate, was obtained from IoLiTec Ionic Liquids Technologies GmbH (Germany). Ultrapure water with a resistance of 18.2 MΩ/cm was obtained using a Direct-Q UV3 system (Merck KGaA, Germany). Ethanol with ≥ 99.5% purity and acetone with ≥ 99.5% purity were from Nacalai Tesque, Inc. (Japan). Oleic acid and its dyestuff oil red were obtained from FUJIFILM Wako Pure Chemical Corporation (Japan). Liquid PDMS (DMS-T05) was obtained from Gelest, Inc. (USA). The food coloring used in Figure 2c was obtained from Kyoritsu Foods Co., Ltd. (Japan). The component ratios were 15% New Coccine and 85% dextrin for red coloring; 14% tartrazine and 86% dextrin for yellow coloring; 8.0% Brilliant Blue FCF and 92% dextrin for blue coloring; and 8.4% Tartrazine, 3.6% Brilliant Blue FCF, and 88% dextrin for green coloring. A black coloring was obtained by mixing all these colorings. Magnetic powder with an average particle diameter of 20 μm (product name: Sr ferrite Type 1) was obtained from Powdertech Co., Ltd. (Japan). The liquid-repellent surface was prepared for picoliter droplet patterning using a solution of an amorphous perfluorinated polymer, Cytop (CTL-809M, AGC Inc., Japan), diluted with CT-Solv.180 (AGC Inc., Japan).

### Particle Preparation

The surface of the fumed titania particles (AEROXIDE TiO_2_ P 90) was modified with silane compounds by chemical vapor deposition. The particles were prefiltered using a metal mesh (obtained from Sanpo Co., Ltd., Japan) with a pore diameter of 63 μm. The filtered particles (1 g) were spread on the bottom of a glass container with a width of 78 mm and height of 105 mm. Then, a 1.5 mL glass bottle loaded with 1 mL of 1H,1H,2H,2H-perfluorotrichlorosilane (or methyltrichlorosilane) was placed inside the glass container (particles were evacuated from the area where the bottle was placed). The glass container was sealed with aluminum foil and heated for 72 h at 120 °C. After heating, the particles were transferred to a light-shielded glass container and cooled to room temperature (20–22 °C).

### Design of Nonsticking Picoliter Droplets

The experimental setup is illustrated in Figure 2a. An ultrasonic spray with a frequency of 40 kHz was obtained using a WS40K ultrasonic spray nozzle and controlled using an AG0001 ultrasonic generator (Sonaer Inc., USA). A commercial hand spray with a container volume of 25 mL was obtained from Artec Co. Ltd. (Japan). A particle powder bed was formed on a glass dish, which was vibrated using a vortex mixer at 1200 rpm (GHW-3000, AS ONE Corporation, Japan).

### Preparation of the Particle-Coated Substrate

A glass slide substrate (Micro slides, Muto Pure Chemicals Co., Ltd., Japan) were coated with a 4 wt % dispersion of the particles in acetone using a spray gun with a nozzle diameter of 0.3 mm (Ausus airbrush 130). The substrates were cleaned for 2 min using a plasma cleaner (PIB-10; Vacuum Device Inc., Japan). The spraying pressure was 0.4 MPa, and the spraying time was approximately 2 s. The density of the coated particles was approximately 2.7 μg/mm^2^.

### Preparation of the Patterned Substrate

Patterned photoactivated surfaces were prepared by masked vacuum ultraviolet (VUV) irradiation of Cytop-coated glass substrates.^61^ A thin layer of Cytop was applied to glass substrates by spin-coating a solution diluted in CT-Solv. 180 at 2,000 rpm for 60 s at 24–25 °C. Subsequently, the Cytop layer was cured at 50 °C and 0.08 MPa for 10 min, 80 °C and 0.02 MPa for 15 min, and 180 °C and 0.02 MPa for 60 min. We used a UV dry processor (PRI03–01, Priways Co., Ltd.) for VUV irradiation using an Xe_2_ excimer lamp. Subsequently, the substrates were placed in a chamber filled with N_2_ gas. A patterned photoactivated polymer surface was produced by masked VUV irradiation through a photomask composed of a predefined Cr mask pattern on a synthesized silica plate.

### Wettability Analysis

All analyses were conducted at room temperature (20–22 °C) and ambient humidity (49–53% RH). The critical surface tensions of the particles were measured using the following protocol. The experimental setup is shown in Figure S1. Ethanol was added to the water until the floating particles detached from the liquid surface. At the point of detachment, the liquid surface tension should be equal to the critical value for complete wetting of the particles by the liquid. We regarded this value as the critical surface tension of the particles. The surface tension was measured using a contact angle meter (Drop Master-SA-Cs1; Kyowa Interface Science Co., Ltd., Japan). We utilized the pendant drop method and processed the samples using Young–Laplace analysis. The static contact, advancing and receding, and tilting angles were also measured using the contact-angle meter. For static contact angle measurement, the probe droplet volume was 10 μL. For the advancing and receding contact angles, we utilized the volume-increasing/decreasing method. The droplet volume variation rate was 1.2 μL/s, and the contact angle images were obtained at 0.1 s intervals. For the tilting angle, the tilting rate was 0.1°/s. The probe liquid was sprayed onto the substrate to measure the sliding angle of a small-volume probe droplet on the coated substrate. The droplet volume was estimated from the sliding-angle images using the ImageJ software.

### Structural Analysis

The particle surface structures were observed using SEM (FE-SEM S-4800; Hitachi High-Technologies Co., Japan). SEM images of the particle-coated glass substrate were obtained by sputtering it with 0.3 nm of Pt. SEM images of the particle-coated picoliter droplets were obtained without pretreatment. The electrostatic droplet motion was recorded using a digital camera (D5600, Nikon Corporation, Japan) with a 105 mm focus lens (AF-S VR Micro-Nikkor 105 mm f/2.8G, Nikon Corporation, Japan) and a teleconverter lens (AF-S TELECONVERTER TC-20E III, Nikon Corporation, Japan). Other photographs and microscopy images were obtained using a digital microscope (DSX-1000, EVIDENT (formerly OLYMPUS) Corporation, Japan).

### Particle Crystallinity Analysis

The crystalline phases were examined by powder X-ray diffraction (XRD; RIGAKU MINIFLEXII) using Cu Kα radiation with Rietveld refinement using SmartLabStudioII. X-ray data were collected in the range of 2θ = 5–120° at 0.02° intervals at room temperature (∼20 °C). We obtained the XRD patterns of the fumed titania particles after heating at 120 °C for 72 h using the same conditions as the particle modification process.

### Particle UV–Vis Light Absorbance Property

The sample was prepared by coating sapphire substrate with a 4 wt % dispersion of the particles in acetone by casting method. The absorbance of the sample was measured using a spectrometer (BIM-6002, Brolight), an integrated sphere (ISP-50–8-R-GT, Ocean Optics), and a UV–Vis light source (L10290, Hamamatsu Photonics K.K.), allowing for the collection of both direct transmittance and forward scattering.

### Surface Chemistry Analysis

The particle surface chemistry was analyzed using the FT-IR attenuated total reflection spectrum obtained from IRSpirit-L (Shimadzu Corp., Japan).

The UV-dependent particle wettability (Figure 4d) was quantified by exposing the particle-coated glass substrate to UV light from the underside of the substrate using a UV spotlight with a wavelength of 365 nm (DSL-365S, Daico Mfg Co., Ltd., Japan). The UV power density was 86 μW/mm^2^, which was measured using a power meter (S120VC, Thorlabs, Inc.) when the uncoated glass substrate passed the light. The area of the UV spot was 27 mm^2^. The substrate was exposed separately to UV light for 1–8 min. Subsequently, the advancing and receding contact angles of the 1-(2-hydroxyethyl)-3-methylimidazolium tetrafluoroborate in the exposed area were measured. We also obtained an FT-IR spectrum (Figure 4c) and SEM image (Figure S11) of the substrate after 30 min of UV exposure and compared them with those of the substrate without UV exposure. UV irradiation (wavelength: 365 nm) was applied under a digital microscope (Figure 4b) using an LED (M365FP1, Thorlabs, Inc.). UV irradiance was measured to be 2.74 mW/mm^2^. For the wetting transition and coalescence experiments (Figure 4e,f), the focused UV irradiation was conducted using a 355 nm laser (LDH-P-FA-355, PicoQuant), and the optical images and movies were captured using a custom-made optical microscope in bright-field mode. The spot size of the UV was ≈ 12 μm.

### Compression Stability

The compression stability of picoliter droplets with a diameter of less than 250 μm was studied using microtweezers (MTW-1E Micro Support Co., Ltd., Japan) under a digital microscope. The compression rate was 11 ± 2 μm/s, and the adhesion of the droplet to the tweezers was used to judge the breakage. Droplets larger than 250 μm were compressed by sandwiching them between two glass substrates. The compression rate for the larger droplets was 77 ± 7 μm/s. The critical compression pressure was estimated using Laplace’s equation *P* = 2γ|cos θ|/*x*, where *x* is the critical compression height at breakage. We assumed that *x* ≪ *D* and θ ≈ 180°.^53^

## Supplementary Material

























## Notes

Particle characterization (surface properties, XRD, UV–Vis, FT-IR, SEM, UV effects); particle-coated droplet properties (friction mechanics, formability, structure, and size tunability); and comparisons of liquid-repellent surfaces (PDF)
Volume-dependent sliding behavior of particle-coated droplets on a glass substrate and uncoated droplets on a particle-coated substrate (MP4)
Compression of a particle-coated picolitre droplet using tweezers (MP4)
Piling behaviors of massive picolitre droplets on a glass substrate and a water pool (MP4)
Picolitre droplet sliding on a water pool under airflow (MP4)
Preprogrammed sliding of picolitre droplets on an oleic acid patterned surface (MP4)
Self-propulsion of picolitre droplets under liquid PDMS (MP4)
Magnetic transportation of picolitre droplets on a glass substrate (MP4)
Electrostatic transportation and assembly of picolitre droplets on a polystyrene substrate (MP4)
UV-triggered sticking transition of picolitre droplets (MP4)
UV-triggered cascade coalescence of picolitre droplets (MP4)
Mechanical splitting of a picolitre droplet into two daughter droplets (MP4)
