# Supplementary material for: Manipulating Pico- to Nanoliter Droplets on Surfaces without Sticking
Source: ACS Nano. 2025 Nov 6;19(45):39398–406. doi: 10.1021/acsnano.5c14919 (PMC12632165; doi:10.1021/acsnano.5c14919)
Supplement: Supplementary file 1 [file nn5c14919_si_001.pdf]

Supporting Information

Manipulating Pico- to Nanoliter Droplets on Surfaces without Sticking

Mizuki Tenjimbayashi[1], Shunto Arai[2], Hiroshi Mizoguchi[1], and Satoshi Ishii[1]*

1. Research Center for Materials Nanoarchitectonics (MANA), National Institute for Materials Science (NIMS)1-1 Namiki, Tsukuba, Ibaraki 305-0044, Japan

2. Research Center for Macromolecules and Biomaterials, National Institute for Materials Science (NIMS)1-1 Namiki, Tsukuba, Ibaraki 305-0044, Japan

*TENJIMBAYASHI.Mizuki@nims.go.jp

Contents

- List of Supplementary Movies
- Table S1. Comparison of the volume-dependent sliding angles on various liquid-repellent surfaces.
- Figure S1. Critical surface tension.
- Figure S2. Surface property of particle-coated glass substrate.
- Figure S3. Structure and friction of non-sticking picolitre droplets.
- Note S1. Volume-dependent droplet sliding angle on various liquid-repellent surfaces.
- Figure S4. Comparison of various liquid-repellent surfaces.
- Note S2. Volume-dependent particle-coated droplet sliding angle on a substrate.
- Figure S5. Volume-dependent sliding angle of particle-coated droplets on a glass substrate.
- Figure S6. Droplet production using hand spray.
- Figure S7. Microscopy images of the particle-coated droplets.
- Figure S8. Formability of the particle-coated droplets.
- Figure S9. XRD patterns of the unmodified fumed titania particles after 72 h at 120 °C.
- Figure S10. UV–VIS absorbance spectrum of the superomniphobic particle.
- Figure S11. FT-IR spectrum of the unmodified particles.
- Figure S12. SEM images of the particle-coated glass substrates before and after UV irradiation.

List of Movies

Movie S1. Volume-dependent sliding behaviour of particle-coated droplets on a glass substrate and uncoated droplets on a particle-coated substrate.

Movie S2. Compression of a particle-coated picolitre droplet using tweezers.

Movie S3. Piling behaviours of massive picolitre droplets on a glass substrate and a water pool.

Movie S4. Picolitre droplet sliding on a water pool under airflow.

Movie S5. Preprogrammed sliding of picolitre droplets on an oleic acid patterned surface.

Movie S6. Self-propulsion of picolitre droplets under silicone oil.

Movie S7. Magnetic transportation of picolitre droplets on a glass substrate.

Movie S8. Electrostatic transportation and assembly of picolitre droplets on a polystyrene substrate.

Movie S9. UV-triggered sticking transition of picolitre droplets.

Movie S10. UV-triggered cascade coalescence of picolitre droplets.

Movie S11. Mechanical splitting of a picolitre droplet into two daughter droplets.

Table S1. Comparison of the volume-dependent sliding angles on various liquid-repellent surfaces.

Probe droplet	Liquid-repellent interface	V [μL]	ρV [mg]	α [°]	Reference
Water	Liquid-like surfaces	10	10	4	29
		10	10	10	
		10	10	13	
		10	10	8	
		50	50	10	30
		40	40	13	
		30	30	14	
		20	20	15	
		10	10	28	
		6	6	41	
		5	5	44	
		4	4	66	

		3	3	8	
		20	20	4	
Diodomethane		3	10	4	
		20	66	2	
Toluene		3	2.6	2	
		20	17	1	
Hexadecane		3	2.3	3	22
		20	15	2	
Cyclohexane		3	2.3	1	
		20	16	1	
Decane		3	2.2	1	
		20	15	1	
Hexane		3	2	1	
		20	13	1	
Hexadecane		14	11	0.37	31
		14	11	0.56	

		14	11	0.97	
		14	11	1.1	
		13	10	0.42	
		13	10	0.58	
		13	10	0.97	
		13	10	1.1	
		12	9.4	0.47	
		12	9.4	0.77	
		12	9.4	1	
		12	9.4	1.5	
		11	8.8	0.45	
		11	8.8	0.59	
		11	8.7	0.59	
		11	8.7	1	
		11	8.8	1.2	
		11	8.7	1.4	

		10	8	0.57	
		10	8	0.61	
		10	8	1.4	
		10	8	1.5	
		9.2	7.1	1.8	
		9.3	7.1	1.3	
		9.3	7.1	0.76	
		9.2	7.1	0.57	
		8.1	6.2	1.9	
		8	6.2	1.5	
		8.1	6.2	1.1	
		8.1	6.2	0.57	
		6.2	4.7	2.3	
		6.1	4.7	2.1	
		6.4	4.9	1.9	
		6.4	4.9	1.7	

		6.1	4.7	1.3	
		6.4	4.9	0.9	
		6.1	4.7	0.79	
		6.4	4.9	0.7	
		5.1	3.9	2.4	
		5.1	3.9	2.2	
		5	3.9	1.3	
		5	3.9	1	
		3.8	2.9	0.93	
		3.8	2.9	1.2	
		3.8	2.9	2.7	
		3.8	2.9	2.7	
		3.1	2.4	2.8	
		3.1	2.4	2.7	
		3.1	2.4	1.6	
		3.1	2.4	1.1	

		1.5	1.2	4.4	
		1.6	1.2	4.4	
		1.5	1.2	3.5	
		1.5	1.2	2.8	
		1.5	1.2	2.7	
Water	Superhydrophobic/omniphobic surfaces	0.023	0.023	Not slide	26[a)]
		0.032	0.032	Not slide	
		0.0075	0.0074	Not slide	
		0.017	0.017	Not slide	
		0.22	0.22	Not slide	
		2.3	2.2	24	26
		2.3	2.2	13	

		2.3	2.2	10	
		2.3	2.2	4	
		2.3	2.2	22	
		0.022	0.022	Not slide	25[a)]
		0.0084	0.0083	Not slide	
		0.0043	0.0043	Not slide	
Ethanol		0.0025	1	Not slide	
Water		50	50	0.8	32
		40	40	0.88	
		30	30	1.3	
		20	20	1.9	
		10	10	3.2	
		5	5	4.3	

		5	5	0.51	33
		0.00066	0.00065	Not slide	23[a)]
		0.000043	0.000043	Not slide	
		0.000077	0.000077	Not slide	
		0.00012	0.00012	Not slide	
		0.00033	0.00033	Not slide	
		0.00033	0.00033	Not slide	
		0.00079	0.00078	Not slide	
		0.00025	0.00025	Not slide	
		0.00053	0.00053	Not	

				slide	
		0.00038	0.00038	Not slide	
		0.0002	0.0002	Not slide	
		0.00055	0.00055	Not slide	
		0.0019	0.0018	Not slide	
		0.0013	0.0013	Not slide	
		0.0013	0.0013	Not slide	
		0.0046	0.0046	Not slide	
Octane		10	7	15	19
Hexadecane	Lubricant-impregnated surface	9.6	7.4	1.6	20
		8	6.2	2	

		5.6	4.3	1.4	
		3.9	3	1.7	
		1.9	1.5	2.2	
Decane		9.5	7	1.8	
		7.8	5.7	2.4	
		6	4.4	2.2	
		2.2	1.6	2.7	
Heptane		6.7	4.6	2	
		1.8	1.2	2.8	
		5.6	3.8	2.1	
		3.5	2.4	2.4	
Ethylene glycol		9.9	11	1.5	
		7.8	8.6	1.8	
		6.2	6.8	1.8	
		4.2	4.6	3	
		2.1	2.3	2.9	

Water		5.8	5.8	2.5	
		3.9	3.9	2.5	
		9.5	9.5	2.2	
		7.8	7.7	2.8	
		2	2	2.3	
		0.065	0.065	Not slide	24
		0.00004	0.00004	Not slide	
		0.000011	0.000011	Not slide	
		0.000044	0.000044	Not slide	
		0.000063	0.000063	Not slide	
		0.000053	0.000053	Not slide	
		0.00003	0.00003	Not	

		2	2	slide	
		0.00004<br>8	0.00004<br>8	Not<br>slide	
		0.00002<br>3	0.00002<br>3	Not<br>slide	
	Hydrophobic surface	28	28	19	34
		27	27	20	
		26	26	21	
		23	23	18	
		22	22	19	
		21	21	21	
		20	20	23	
		19	19	25	
		17	17	21	
		16	16	21	
		15	15	23	

		14	14	25	
		14	14	22	
		13	13	24	
		13	13	27	
		11	11	26	
		10	10	29	
		12	12	31	
		11	11	33	
		29	29	17	
		28	28	17	
		27	27	19	
		26	26	19	
		24	24	17	
		23	23	18	
		22	22	18	
		21	21	19	

		19	19	22	
		18	18	22	
		17	17	23	
		15	15	21	
		14	14	22	
		33	33	16	
		32	32	18	
		27	27	17	
		26	26	18	
		22	22	24	
		21	21	25	
		18	18	23	
		20	20	29	
		17	17	27	
		16	16	32	
		33	33	19	

		32	32	20	
		27	27	19	
		26	26	20	
		22	22	25	
		21	21	26	
		18	18	24	
		17	17	27	
		20	20	29	
		16	16	32	
	Liquid marble	50	50	2.6	32
		40	40	2.5	
		30	30	2.7	
		20	20	2.7	
		10	9.9	2.7	
		5.1	5.1	2.6	

a) We regard the Wenzel state droplet as “not slide”.

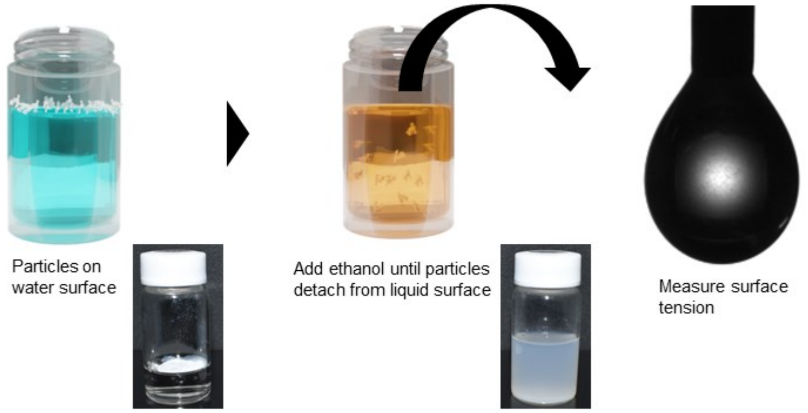

Figure S1. Critical surface tension. Method used to measure the critical surface tension of the particles.

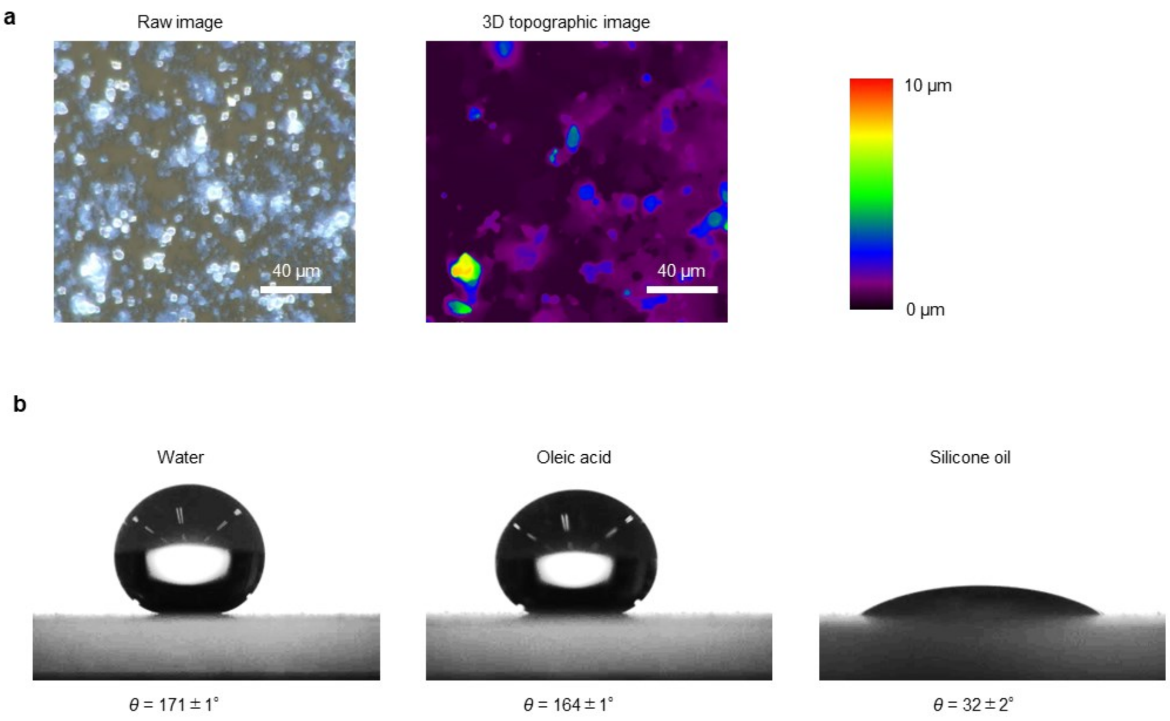

Figure S2. Surface property of particle-coated glass substrate. a, Surface microscopy images and **b**, photos of 10 μL droplets resting on the particle-coated glass substrate.

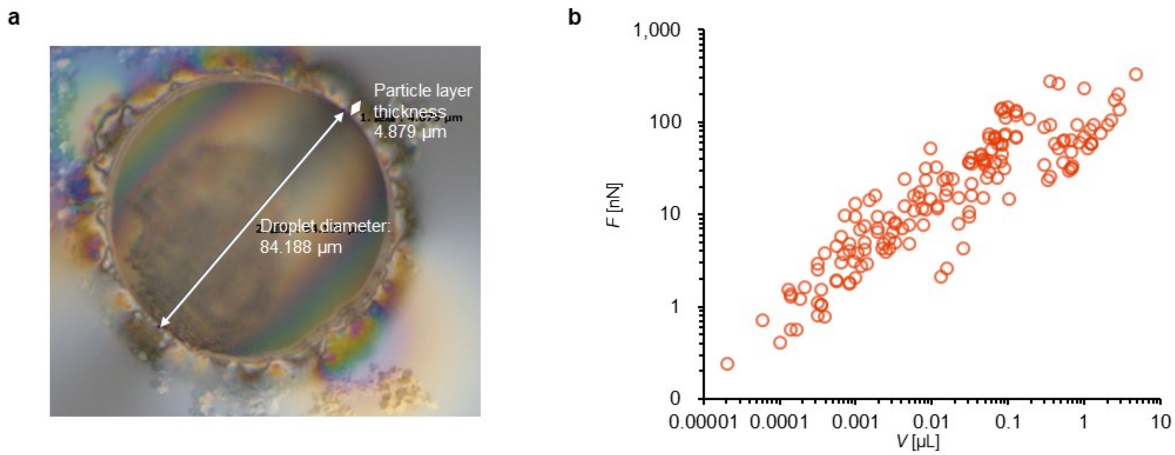

Figure S3. Non-sticking picolitre droplets. a, Typical thickness of the particle layer on the picolitre droplets. **b**, Gravitational force required to cause the non-sticking droplets to start sliding as a function of droplet volume.

Note S1. Volume-dependent droplet sliding angle on liquid-repellent surfaces.

The droplets begin sliding under the action of the gravitational force $Fg = \rho Vg\sin\alpha$ where ρ is the liquid density, V is the apparent droplet volume, and g is gravitational acceleration. On liquid-repellent coated surfaces, the force is balanced mainly by droplet contact line friction, which is approximately $\gamma_d\Phi_{SL}D$, where γ_d, D are the droplet surface tension, diameter and Φ_{SL} is the solid−liquid contact fraction. Assuming that the droplet has a spherical cap shape, then $V = 24^{-1}\pi D^3(2 + \cos\theta)(2 - \cos\theta)^2$. Thus, the effect of the droplet volume on the sliding angle can be expressed as $\sin\alpha \approx c_{SL}V^{-2/3}$, where $c_{SL} = 24^{1/3}\pi^{-1/3}\gamma_d\Phi_s\rho^{-1}g^{-1}[(2 + \cos\theta)(2 - \cos\theta)^2]^{-1/3}$. This explains why the droplet sliding angle increases exponentially as the droplet volume decreases, as shown in our experiment. **Figure S4** compares the volume-dependent droplet sliding angles on liquid-repellent surfaces obtained from **Table S1**, expressed as a log–log plot with a slope of −2/3.

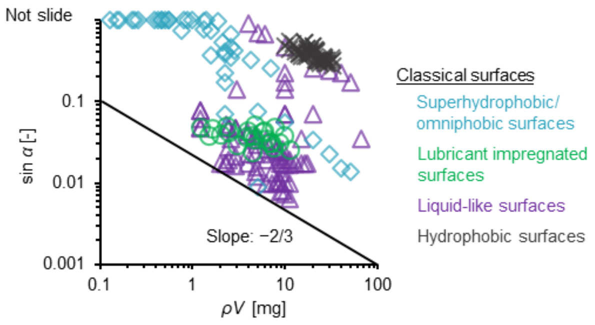

Figure S4. Comparison of various liquid-repellent surfaces. In addition to the data from this work, data were obtained from previous works (see Table S1 for details).

Note 2. Volume-dependent particle-coated droplet sliding angle on a substrate.

The particle-coated droplets behaved like soft solids. As a primary model, we consider the particle-coated picolitre droplets as solid spheres, despite the fact that they are not homogeneous. In this state, friction is caused by solid–solid interactions between the particles and the substrate, rather than the contact line. We consider the solid−solid friction working to resist gravity as $F = \mu N$, where N is the normal force, which balances gravity and probably van der Waals interactions, which can be expressed as $N = \rho Vg\cos\alpha + f_{vdW}D$. Balancing with the gravity and the friction, we obtain $\sin\alpha = \mu\cos\alpha + \mu(\rho Vg)^{-1} f_{vdW}D$. At high droplet volumes (millimetric droplets), the van der Waals factor is negligible, and we obtain the classical friction law: $\sin\alpha = (1 + \mu^{-2})^{-1/2}$.[37] However, when the droplet volume decreases and the range of the applied force for sliding is less than a single nanonewton, the van der Waals factor becomes important. The van der Waals factor is expected to dominate the friction and $\sin\alpha \approx \mu(\rho Vg)^{-1}f_{vdW}D$. Thus, we obtain $\sin\alpha \approx c_{SS}V^{-2/3}$, where $c_{SS} = 24^{1/3}\pi^{-1/3}\mu f_{vdW}\rho^{-1}g^{-1}[(2 + \cos\theta)(2 - \cos\theta)^2]^{-1/3}$. Hence, the observed sliding angle increased as the volume decreased, and the asymptote slope is within the range of −2/3–0 and increases with V, which is consistent with the data in **Figure S5**.

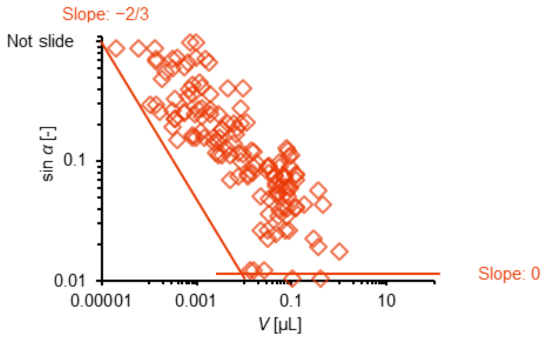

Figure S5. Volume-dependent sliding angle of the particle-coated droplets on a glass substrate.

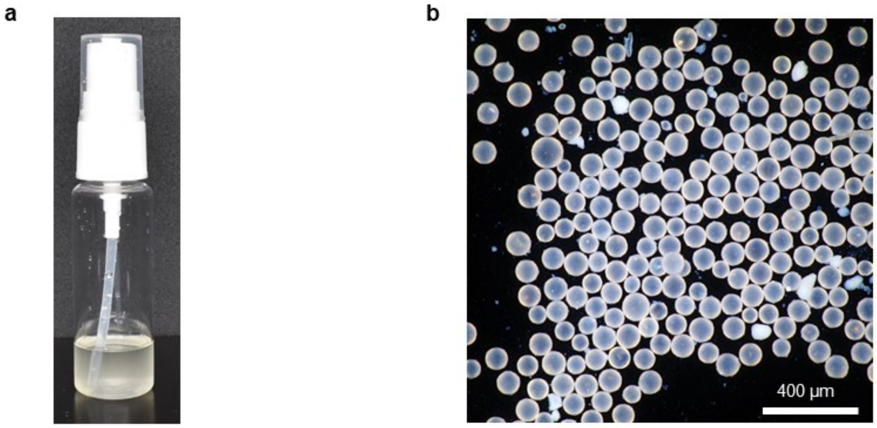

Figure S6. Droplet production using hand spray. a, Production of non-sticking droplets using hand spray and **b**, microscopy images of the prepared droplets.

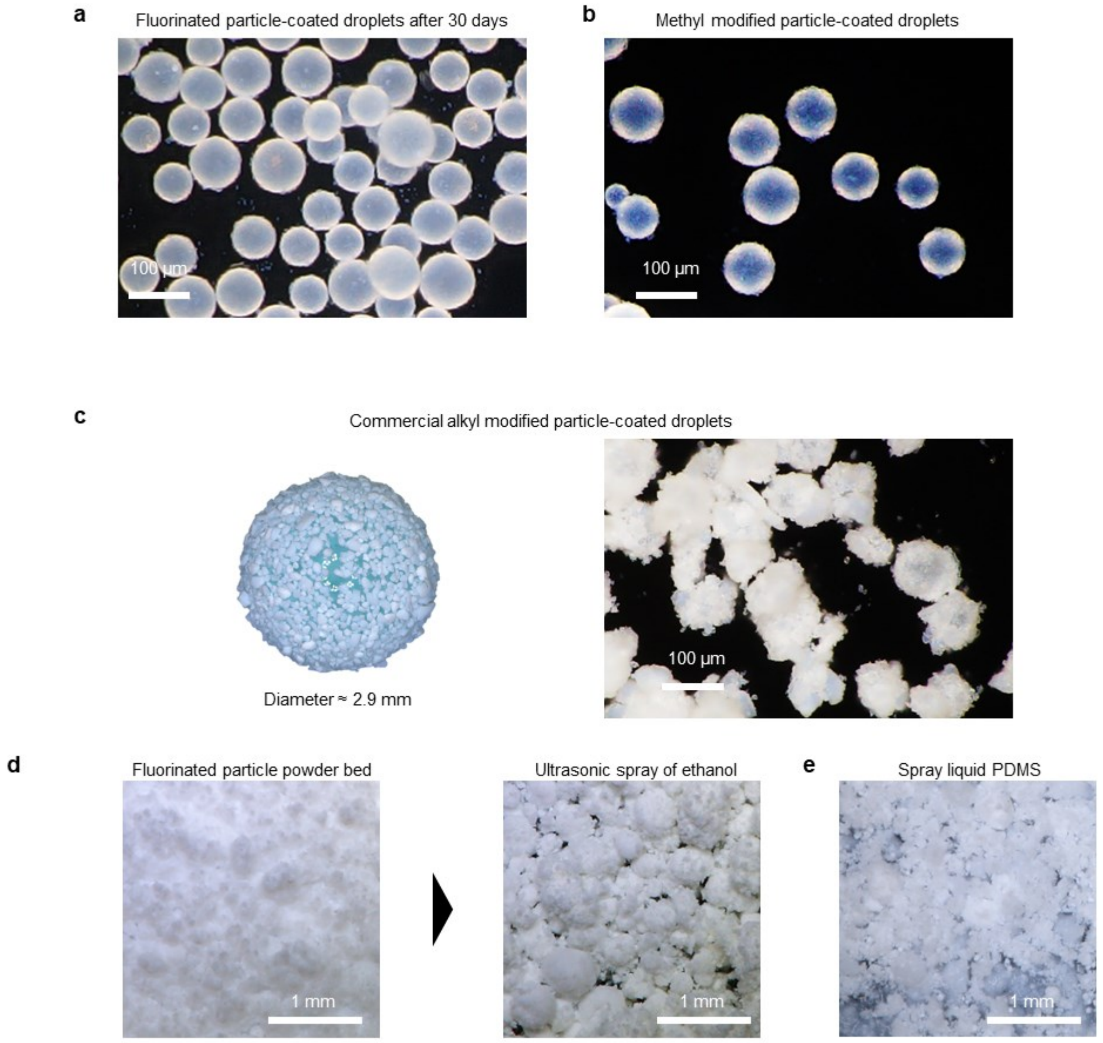

Figure S7. Formability of the particle-coated droplets. Droplets coated with **a**, fluorinated particle-coated droplets after 30 d in a glass bottle and **b**, methyl-modified particles. **c**, Commercial alkyl-modified particles used for coating millimetric droplet (Left). However, the coating is failed for ultrasonic sprayed droplets (Right). **d**, Powder bed images of the fluorinated particle before and after spraying of ethanol and liquid PDMS.

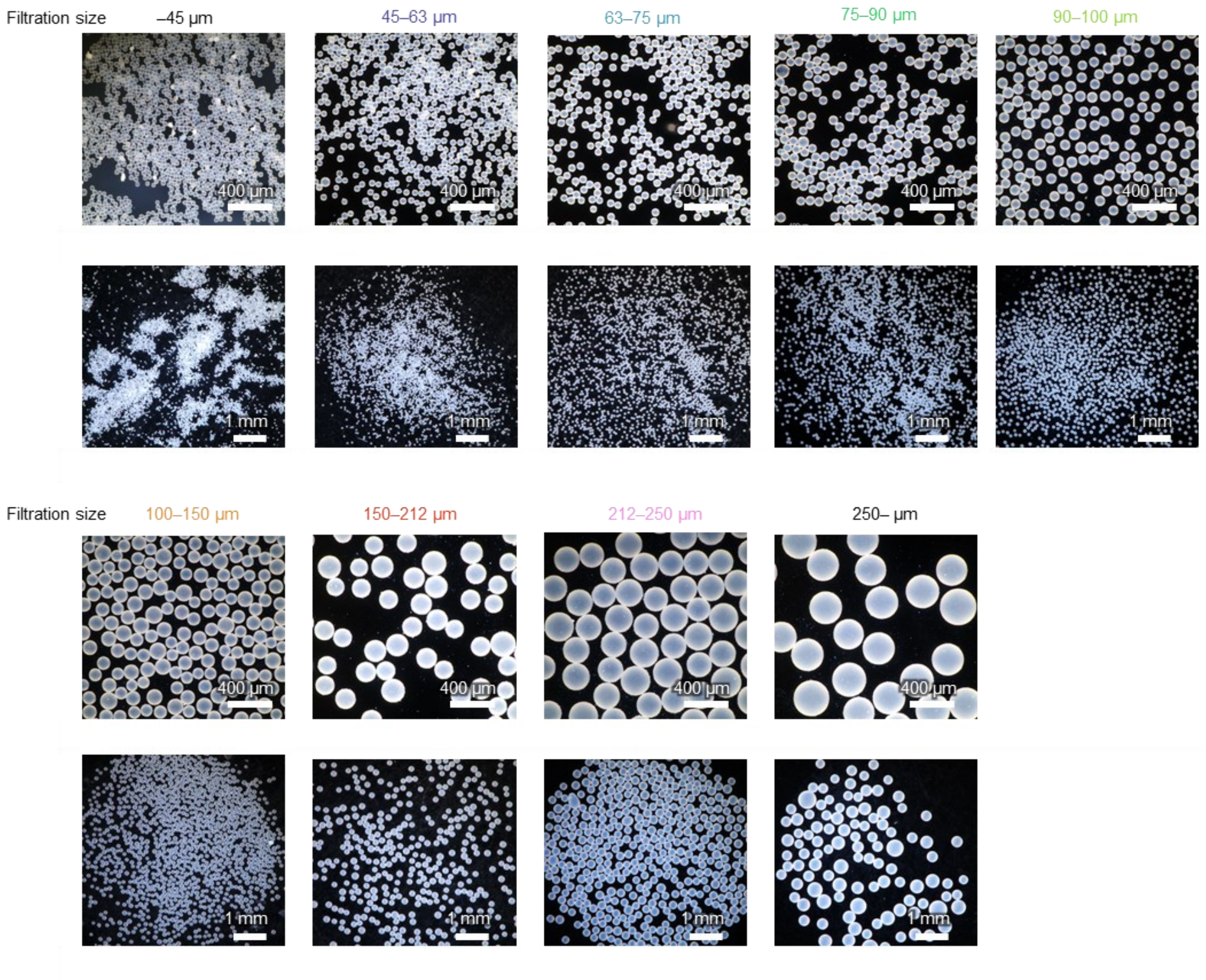

Figure S8. Different droplet sizes. Microscopy images of particle-coated droplets of different sizes after filtration.

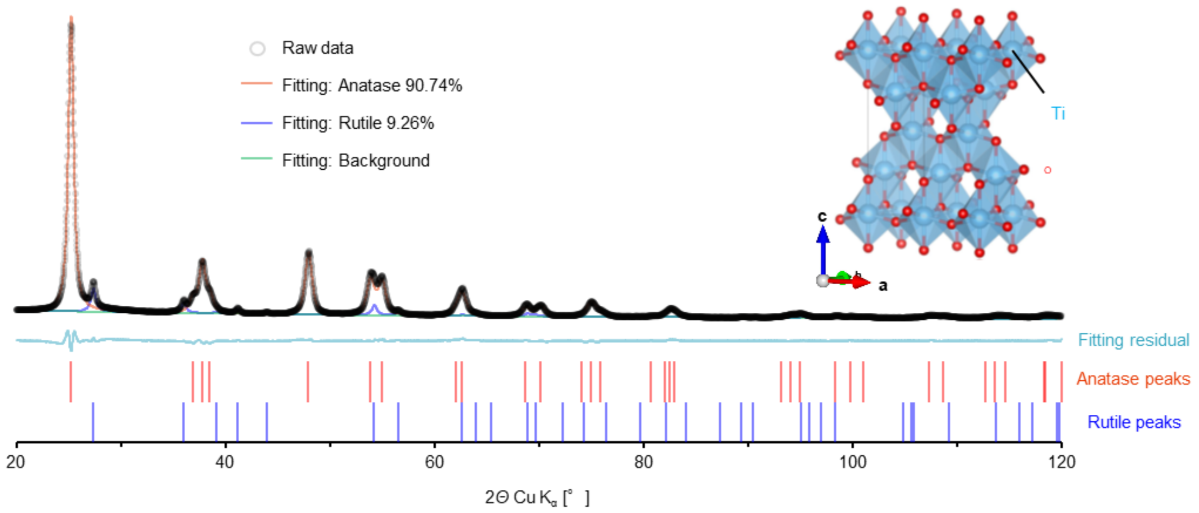

Figure S9. XRD patterns of the unmodified fumed titania particles after 72 h at 120 °C. The black dots indicate the measured data. The red and blue lines indicate peak positions. The fitting results for the anatase and rutile phases were obtained using Rietveld refinements. Inset: crystal structure of anatase TiO_2.

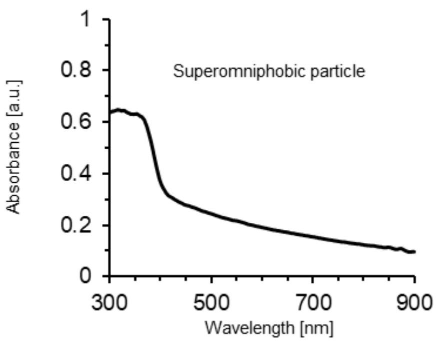

Figure S10. UV–VIS absorbance spectrum of the superomniphobic particle.

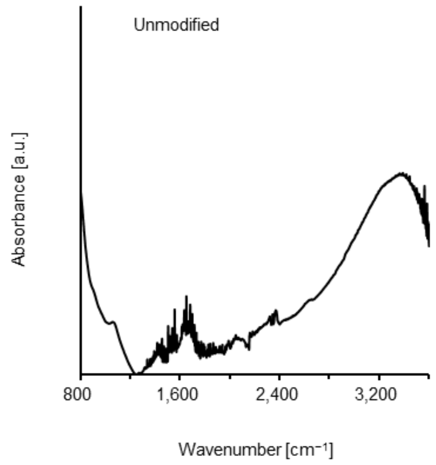

Figure S11. FT-IR spectrum of the unmodified particles.

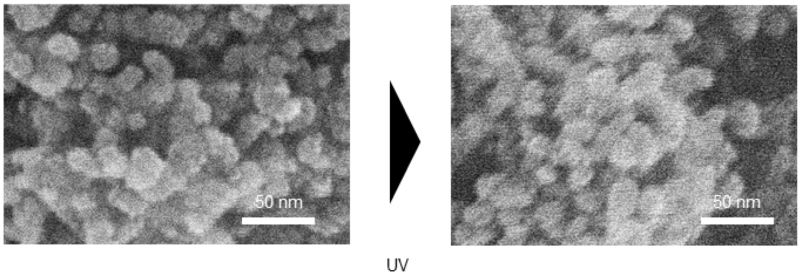

Figure S12. SEM images of the particle-coated glass substrates before and after UV irradiation.
